# Magnesium Supplement and the 15q11.2 BP1–BP2 Microdeletion (Burnside–Butler) Syndrome: A Potential Treatment?

**DOI:** 10.3390/ijms20122914

**Published:** 2019-06-14

**Authors:** Merlin G. Butler

**Affiliations:** Departments of Psychiatry & Behavioral Sciences and Pediatrics, University of Kansas Medical Center, Kansas City, KS 66160, USA; mbutler4@kumc.edu; Tel.: +1-913-588-1800

**Keywords:** 15q11.2 BP1–BP2 microdeletion (Burnside–Butler syndrome), *NIPA1*, *NIPA2*, *CYFIP1*, *TUBGCP5* genes, Prader–Willi and Angelman syndromes, magnesium transporters and supplementation, potential treatment options

## Abstract

The 15q11.2 BP1–BP2 microdeletion (Burnside–Butler) syndrome is an emerging disorder that encompasses four genes (*NIPA1, NIPA2, CYFIP1*, and *TUBGCP5*). When disturbed, these four genes can lead to cognitive impairment, language and/or motor delay, psychiatric/behavioral problems (attention-deficit hyperactivity, autism, dyslexia, schizophrenia/paranoid psychosis), ataxia, seizures, poor coordination, congenital anomalies, and abnormal brain imaging. This microdeletion was reported as the most common cytogenetic finding when using ultra-high- resolution chromosomal microarrays in patients presenting for genetic services due to autism with or without additional clinical features. Additionally, those individuals with Prader–Willi or Angelman syndromes having the larger typical 15q11–q13 type I deletion which includes the 15q11.2 BP1–BP2 region containing the four genes, show higher clinical severity than those having the smaller 15q11–q13 deletion where these four genes are intact. Two of the four genes (i.e., *NIPA1* and *NIPA2*) are expressed in the brain and encode magnesium transporters. Magnesium is required in over 300 enzyme systems that are critical for multiple cellular functions, energy expenditure, protein synthesis, DNA transcription, and muscle and nerve function. Low levels of magnesium are found in those with seizures, depression, and acute or chronic brain diseases. Anecdotally, parents have administered magnesium supplements to their children with the 15q11.2 BP1–BP2 microdeletion and have observed improvement in behavior and clinical presentation. These observations require more attention from the medical community and should include controlled studies to determine if magnesium supplements could be a treatment option for this microdeletion syndrome and also for a subset of individuals with Prader–Willi and Angelman syndromes.

## 1. Introduction

**Figure 1:**
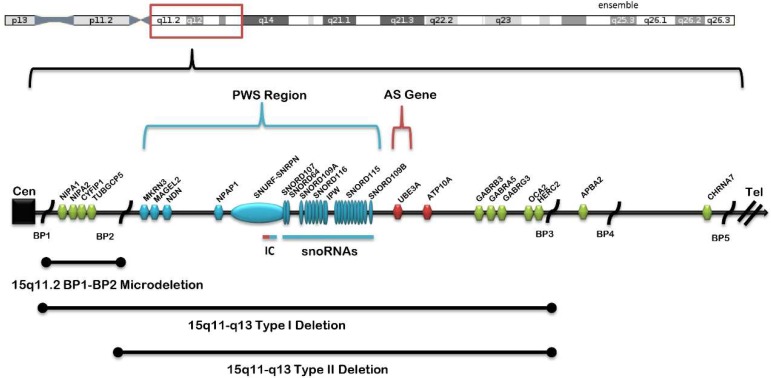
Chromosome 15 ideogram showing the location of genes and transcripts causing Prader–Willi syndrome (PWS) that are imprinted and paternally expressed (blue) and Angelman syndrome (AS) which are imprinted and maternally expressed (red). The location and size of the 15q11.2 BP1–BP2 microdeletion, the typical larger 15q11–q13 type I deletion involving breakpoints BP1 and BP3, and the typical smaller 15q1–q13 type II deletion involving breakpoints BP2 and BP3 are illustrated. IC: imprinting center controlling the activity of imprinted genes in the 15q11–q13 region.

Clinical and behavioral differences have been reported over the past 15 years in Prader–Willi syndrome (PWS) and Angelman syndrome (AS), with the identification of specific molecular classes [1,2]. PWS and AS were the first examples of errors in genomic imprinting in humans [2,3,4,5], although they are entirely different clinical disorders. The most frequent genetic defect is a deletion of the paternal chromosome 15q11–q13 region in PWS or of the same maternal chromosome 15 region in in AS. The typical 15q11–q13 deletions are classified as either type I, involving the proximal 15q breakpoint BP1 and the distal 15q breakpoint BP3, or type II, involving the proximal 15q breakpoint BP2 and BP3 in both syndromes [2,5,6,7,8,9] (see Figure 1). The larger type I deletion is approximately 6.6 Mb in size and includes four genes (*TUBGCP5*, *CYFIFP1*, *NIPA*, and *NIPA2*) located in the 15q11.2 BP1–BP2 region, while the smaller type II deletion is 5.3 Mb in size and leaves the four genes intact [10]. Individuals with PWS or AS and the larger type I deletion often have increased learning, behavioral, or clinical problems compared to those with the smaller typical type II deletion [1], specifically, more frequent compulsions, self-injury episodes, and maladaptive behaviors with lower cognitive, reading, and math skills than PWS patients with type II deletions and more impaired speech and seizure activity than AS patients with the smaller deletion.

## 2. Background and Significance

The now recognized 15q11.2 BP1–BP2 microdeletion (Burnside–Butler) syndrome involves only four genes in the region and can present with cognitive impairment, language and/or motor delay, autism, behavioral problems, poor coordination, ataxia, and congenital anomalies but not with AS or PWS. Psychiatric findings can include schizophrenia, oppositional defiant disorder, obsessive compulsive disorder, dyslexia, and structural brain defects [11,12,13,14]. Greater than two-thirds of individuals with this microdeletion present with a range of recognized clinical findings, but most individuals have not been assessed with detailed clinical, behavioral, and advanced genetic testing to identify features that may be related to the syndrome. 

This emerging microdeletion syndrome encompasses the region between the proximal 15q breakpoints BP1 and BP2 including *TUBGCP5*, *CYFIP1*, *NIPA1*, and *NIPA2* genes, with an estimated prevalence from 0.6 to 1.3% based on early studies in patients presenting with unexplained behavior, cognitive, and/or psychiatric problems [11,15,16,17,18,19]. Later, Ho et al. [20] summarized the results of over 10,000 consecutive patients presenting with autism spectrum disorder with or without congenital anomalies or other problems using ultra-high microarray analysis and found this microdeletion to be the most common cytogenetic finding.

Cox and Butler [11] reviewed 200 individuals with the 15q11.2 BP1–BP2 microdeletion reported in the literature and grouped the findings into five categories: (1) developmental (73% of cases), speech (67%), and motor delays (42%); (2) dysmorphic ears (46%) and palatal anomalies (46%); (3) writing (60%) and reading (57%) difficulties, memory problems (60%), and verbal IQ scores ≤75 (50%); (4) general behavioral problems, unspecified (55%); and (5) abnormal brain imaging (43%). Other less frequent features observed were seizures/epilepsy (26%), autism spectrum disorder (27%), attention-deficit hyperactivity disorder (ADHD, 35%), and schizophrenia/paranoid psychosis (20%). The four genes in the 15q11.2 BP1–BP2 region are highly conserved and include *NIPA1* (non-imprinted in PWS and AS1) which causes autosomal dominant hereditary spastic paraplegia (SPG1) and postural disturbances [21,22]. An association of *NIPA1* gene repeat expansions with amyotrophic lateral sclerosis has been reported with some features in common with the 15q11.2 BP1–BP2 microdeletion syndrome [23]. This gene is known to mediate Mg^2+^ transport and is highly expressed in the brain [24]. *NIPA1* can also transport other divalent cations such as Fe^2+^, Sr^2+^, Ba^2+^, Mn^2+^, and Co^2+^ but to a lesser extent. *NIPA2* is highly selective as a magnesium transporter. Three specific *NIPA2* mutations (p.I178F, p.N244S, and p.N334_E335insD) have been reported in patients with childhood absence epilepsy [24]. Functional analysis of the mutant *NIPA2* gene variants showed decreased intracellular magnesium concentration in neurons, suggesting that lower intracellular magnesium concentrations would enhance *N*-methyl-d-aspartate receptor (NMDAR) currents and impact neuron excitability and brain function. Mutant proteins were not trafficked adequately to the cell membrane for normal function [24]. 

Magnesium transport involves the binding of fully hydrated cations to an extracellular binding loop connecting transmembrane domains and their passage through the cellular membrane without electrostatic interactions [25]. Magnesium is required for over 300 enzyme systems that are critical for multiple cellular functions, energy expenditure—including oxidative phosphorylation and glycolysis—as well as DNA transcription, protein synthesis, and muscle and nerve function. Some studies have suggested that the modern Western diet may lead to magnesium deficiency which is associated with a wide range of medical conditions including constipation, sleep disturbances, epilepsy, muscle cramps, and depression [26]. The recommended dietary allowance is 80 mg at 1–3 years of age; 130 mg at 4–8 years; 240 mg at 9–13 years, and 400 mg during adulthood. Common sources of magnesium include whole grain, spinach, almonds, peanuts, cashews, avocados, dark chocolate, and black beans. 

## 3. Discussion

The magnesium ion fulfils several important functions in living organisms and is unique amongst the biological cations. Magnesium transporters are found in prokaryotes, but a group of newly identified transporters, including TRPM6/7, NIPA2, MagT, MMgT, and HIP14, are not found in prokaryotes. However, these mammalian magnesium transporters have no obvious amino acid similarities, indicating there are many ways to transport Mg^2+^ across membranes by using a wide variety of structural properties and physiological functions [27]. Recent studies have shown the critical role of two magnesium transporters (i.e., TRPM6 and TRPM7) that belong to the transient receptor potential (TRP) family. Transient receptor potential melastatin 7 (TRPM7) is one of these cellular receptors that mediate the entry of extracellular Mg^2+^ into cells and is required for cellular magnesium homeostasis. TRPM6 also plays a role as an epithelial magnesium transporter, and loss-of-function *TRPM6* gene mutations are found in those with a severe form of hereditary hypomagnesaemia. Both of these receptors have an atypical kinase domain that functions in the role of a transporter [28]. 

When disturbed, many identified Mg^2+^ transporters are also associated with congenital disorders encompassing a wide range of tissues including kidney, brain, intestine, and skin [27]. Magnesium is a potential modulator of seizure activity because of its ability to antagonize excitation of the NMDA receptors [26]. Magnesium deficiency causes NMDA-coupled calcium channels to be biased towards opening, thereby causing neuronal injury and neurological dysfunctions such as major depression [29]. Magnesium supplementation may be effective in treating depression. In addition, magnesium levels are reduced in both acute and chronic brain diseases. This has raised an interest in examining the role of magnesium in normal and injured nervous system, possibly involving the two main barrier systems. These barrier systems are the blood–brain barrier formed by brain capillary endothelial cells, which separates blood from extracellular fluid, and the blood–cerebral spinal fluid (CSF) barrier formed by choroidal epithelial cells, which separates blood from CSF. How magnesium transport takes place between the blood–brain and blood–CSF barriers is not clearly understood. It is evident that magnesium enters the brain through the blood–brain barrier and is actively transported by choroidal epithelial cells into the CSF. These epithelial cells express both *TRPM6* and *TRPM7* genes and may play a role in magnesium transport into the central nervous system [30].

Most Mg^2+^ transporter proteins transport a number of divalent cations besides Mg^2+^ across membranes, but NIPA2 is one of the few magnesium transporters that is selective for magnesium. Recent studies have shown that the absence of NIPA2 enhances neural excitability through the BK potassium channels, as evidenced from experiments in NIPA2 knockout mice versus wild-type mice using whole-cell patch-clamp recordings to measure the electrophysiological properties of neocortical somatosensory pyramidal neurons [31]. Magnesium also induces neuronal apoptosis by suppressing excitability, but the effects on brain development are unknown [32]. Information learned in these studies might lead to a better understanding of central nervous system disturbances in individuals with specific magnesium transporter gene defects. For example, *NIPA2* gene defects cause childhood epilepsy and possibly play a role in the phenotype of the 15q11.2 BP1–BP2 deletion syndrome. Patients with AS having the larger 15q11–q13 type I deletion including the four genes in the 15q11.2 BP1–BP2 region (and both *NIPA1* and *NIPA2*) are more severely affected, with increased seizure activity. Studies have shown that people with epilepsy have lower magnesium levels than individuals without epilepsy. In addition, a poor magnesium status is also recognized as a risk factor for Alzheimer’s disease, but the underlying mechanism is unclear. Elevated magnesium reduces blood–brain barrier permeability and accelerates the clearance of amyloid beta peptide from the brain [33].

Recently, MgSO_4_ and magnesium transporters were studied to determine if they have a protective effect on neurotoxicity induced in living cells. Cells that were chemically stressed and then treated with MgSO_4_ showed improved viability and increased cellular mRNA for the protein encoded by the *NIPA1* gene located in the 15q11.2 BP1–BP2 region. These results did suggest that MgSO_4_ may have a protective effect and that NIPA1 protein might be involved in dopaminergic neurons [34]. The *NIPA1* gene encodes a magnesium transporter protein located in early endosomes and at the cell surface of neurons. In addition, Chang et al. [35] proposed that targeting Mg^2+^ uptake mediated by NIPA1 may be an interesting option for novel therapies for conditions such as anorexia nervosa associated with 15q11.2 BP1–BP2 microduplications and other neurobehavioral disorders. A second gene in the 15q11.2 BP1–BP2 region is *NIPA2* (non-imprinted in PWS and AS2), which also encodes a protein that plays a role in magnesium transport in renal cells with mutations causing childhood absence epilepsy [36]. The *TUBGCP5* (tubulin gamma complex-associated protein 5) gene in this region is associated with ADHD and obsessive-compulsive disorder when disturbed [16]. The fourth gene is *CYFIP1* (cytoplasmic fragile X mental retardation 1 FMR1-interacting protein 1) whose protein product interacts with FMRP, the protein coded by the *FMR1* gene. Mutations of *FMR1* lead to fragile X syndrome and now recognized as the leading cause of familial intellectual disability [37].

The 15q11.2 BP1–BP2 microdeletion syndrome was found in 9% of the top 85 microarray cytogenetic results in a recent study reported by Ho et al. [20] in a large cohort of patients presenting for genetic services. However, there is a lack of detailed neuropsychiatric and behavior assessments in family members (and parents) of those with the microdeletion. More sophisticated genetic testing is now available to better characterize the relationship between the cytogenetic defect and clinical presentation, variability, and severity. Additional testing may include next-generation (exome) sequencing or targeted approaches to analyze the four genes in the 15q11.2 BP1–BP2 region and determine if there is a variant of one of the non-deleted genes in the region leading to a more severe phenotype or associated with interactive genes outside of the region but in common biological pathways. 

Further research with combined behavior/psychiatric/cognitive/motor measures are needed of “affected” and “non-affected” family members with and without the microdeletion. This would allow the determination of the coding genetic status of the “normal” or “non-deleted” candidate gene alleles in families. In addition, clinical description and phenotypic findings may be incomplete, poorly characterized, or unavailable for parents and other family members having the microdeletion, without concerted efforts to undertake such testing and detailed assessments to thoroughly address these issues and examine genotype–phenotype relationships. Currently, about one-third of children inherit the microdeletion from an abnormal parent. Hence, modified genes outside of this chromosome region may also play a role but require further investigations, as suggested by examining interacting genes or pathways impacting on the phenotype and clinical presentation.

Aberrant behavior including aggression is one of the major problems that parents face with children having this microdeletion syndrome. Two of the four deleted genes in the region (i.e., *NIPA1* and *NIPA2*) encode magnesium transporter proteins and were targeted by several families exchanging information via internet communication. They administered magnesium supplements to their affected children for treatment. Their efforts were supported in the literature by Chang et al. [35] in investigations that proposed that Mg^2+^ therapy may have a positive impact. Magnesium taurate and magnesium L-threonate were used historically in anecdotal reports to improve behavior. Additionally, selenium, as an antioxidant, was also used by parents to improve mental alertness or calmness in their affected children, but magnesium with a general vitamin regime was the most effective in improving behavior, according to the parents. High iron levels reported by parents in some of their children were also lowered when magnesium taurate was introduced into the diet. Other patients showed improved behavior when administered magnesium l-threonine. 

Although anecdotally, the above information from families requires more attention and investigation to address these early observations linking magnesium supplementation and improved behavior in those subjects with the 15q11.2 BP1–BP2 microdeletion. Two of the four genes in this chromosome region are involved in the transportation of magnesium and, to a lesser degree, of other cations. This information, if true, could also be applied to behavioral problems seen in PWS and AS with the larger 15q11–q13 type I deletions. Those PWS and AS patients with the larger deletion present higher clinical severity and are missing the two magnesium transporter genes in the 15q11.2 BP1–BP2 region. Controlled studies with magnesium supplementation and behavioral measures in a large cohort of subjects with the 15q11.2 BP1–BP2 microdeletion are needed to further investigate these observations.
